# Cluster analysis dissecting cognitive deficits in older adults with major depressive disorder and the association with neurofilament light chain

**DOI:** 10.1186/s12877-024-04960-z

**Published:** 2024-04-16

**Authors:** Cynthia Yi-an Chen, Chih-Chiang Chiu, Cho-Yin Huang, Ying-Chih Cheng, Ming-Chyi Huang, Po-Hsiu Kuo, Wen-Yin Chen

**Affiliations:** 1 Department of Psychiatry, Wan-Fang Hospital, Taipei Medical University, Taipei, Taiwan; 2 Department of Psychiatry, Taipei City Psychiatric Center, Songde branch, Taipei City Hospital, Taipei, Taiwan; 3 Institute of Epidemiology and Preventive Medicine, College of Public Health, National Taiwan University, Taipei, Taiwan; 4 Department of Psychiatry, School of Medicine, College of Medicine, Taipei Medical University, Taipei, Taiwan; 5 Department of Psychiatry, China Medical University Hsinchu Hospital, China Medical University, Hsinchu, Taiwan; 6 Department of Public Health, College of Public Health, National Taiwan University, Taipei, Taiwan; 7 School of Medicine, College of Medicine, Fu Jen Catholic University, New Taipei, Taiwan

**Keywords:** Major depressive disorder, Cognitive impairment, Dementia, Neurofilament light chain, Cluster analysis

## Abstract

**Background:**

Cognitive impairment is a growing problem with increasing burden in global aging. Older adults with major depressive disorder (MDD) have higher risk of dementia. Neurofilament light chain (NfL) has been proven as a potential biomarker in neurodegenerative disease, including dementia. We aimed to investigate the association between cognitive deficits and NfL levels in older adults with MDD.

**Methods:**

In this cross-sectional study, we enrolled 39 MDD patients and 15 individuals with mild neurocognitive disorder or major neurocognitive disorder, Alzheimer’s type, as controls, from a tertiary psychiatric hospital. Both groups were over age 65 and with matched Mini-Mental State Examination (MMSE) score. Demographic data, clinical variables, and plasma NfL levels were obtained. We used cluster analysis according to their cognitive profile and estimated the correlation between plasma NfL levels and each cognitive domain.

**Results:**

In the MDD group, participants had higher rate of family psychiatry history and current alcohol use habit compared with controls. Control group of neurocognitive disorders showed significantly lower score in total MMSE and higher plasma NfL levels. Part of the MDD patients presented cognitive deficits clustered with that of neurocognitive disorders (cluster A). In cluster A, the total MMSE score (*r*=-0.58277, *p*=0.0287) and the comprehension domain (*r*=-0.71717, *p*=0.0039) were negatively correlated to NfL levels after adjusting for age, while the associations had not been observed in the other cluster.

**Conclusions:**

We noted the negative correlation between NfL levels and cognition in MDD patients clustered with neurodegenerative disorder, Alzheimer’s type. NfL could be a promising candidate as a biomarker to predict subtype of patients in MDD to develop cognitive decline. Further longitudinal studies and within MDD cluster analysis are required to validate our findings for clinical implications.

## Introduction

Cognitive impairment is a growing problem with increasing burden in aging global population. The estimated prevalence of people with dementia would reach 83.2 million in 2030 and 152.8 million in 2050, affecting 23.5% of men and 30.5% of women who are 85 years and older [1]. There were around 28.8 million disability-adjusted life-years attributed to dementia [2]. Older adults with major depressive disorder (MDD) have higher risk of dementia as compared with general population during aging [3–5]. Previous analyses estimated an odd ratio ranged from 1.78 to 2.11 for people with history of depression to develop Alzheimer disease (AD) [6]. In addition, increased severity of depression also seemed to increase the risk of incident dementia [7].

In patients with MDD, cognitive impairment is a common symptom which is related to poorer outcomes [8]. Patients in depressive episode presented impairment of memory, executive function, and attention comparing to non-depressed population [9]. Residual cognitive problem might persist despite the remission of depressive symptoms [9, 10], especially in the domains of executive function and attention [9]. In addition, studies in older patients with depression have revealed similar results [11, 12]. Reduced hippocampal volume was found in some of the older patients with depression [12–14], which might implicate part of the underlying mechanism for cognitive impairment.

Studies in biomarkers related to depression focusing on their value to predict cognitive function are less abundant [15]. Hypercortisolemia has been found to be associated with impaired cognitive function in both younger and older depressed persons [12, 16]. Studies using the (18)F-florbetapir (AV-45/Amyvid) positron emission tomography (PET) had already suggested that the subgroup of late life MDD patients with high amyloid burden implied poorer memory performance and higher risk to develop AD [17, 18]. Peripheral biomarkers related to cognitive decline has become an important study field in recent years. Neurofilament light chain (NfL) has been one of the potential peripheral biomarkers related to neurodegenerative diseases [19]. NfL is a 61.5kDa cytoskeletal protein which is exclusively expressed in neurons [19]. NfL can be detected in cerebrospinal fluid (CSF) and peripheral blood, though with a much lower concentration in the latter. Elevation of NfL levels indicates neuroaxonal injury, which might be found in normal aging but also in neurodegenerative diseases, such as dementia, multiple sclerosis, Parkinson’s disease, and Huntington disease [19]. The current digital immunoassay technology provided higher sensitivity and thus a reliable method to detect plasma concentration of NfL [19]. It offered an easier way to monitor longitudinal changes without the need to obtain CSF specimen. Recent studies have revealed the implications of peripheral NfL levels to predict the progression to dementia from mild neurocognitive impairment (MCI) [20–22].

In this study, we first clarified the cognitive profile of older MDD participants as compared with older participants with MCI [23] or AD, which is the most common type of dementia [24, 25]. Then, we aimed to dissect the cognitive profile by cluster analysis, to confirm that a subgroup of older MDD patients with similar cognitive profile as MCI or AD would present a correlation between NfL and their cognitive manifestation.

## Methods

### Participants

This is a case-control study enrolling participants from January 2020 to July 2021 from outpatient department in a tertiary psychiatric hospital. We enrolled participants diagnosed with MDD according to Diagnostic and Statistical Manual of Mental Disorders, Fifth Edition (DSM-5) criteria and they were aged 65 or over. They also fulfilled the following inclusion criteria: 1) Mini-Mental State Examination (MMSE) score was between 18- 30 [26, 27]; 2) not in an acute depressive episode, measured as 17-item Hamilton Depression Rating Scale (HAMD-17) less than 16 [28]. Participants were further excluded if they were comorbid with substance use disorder or other active physical conditions which may threaten their lives or had comorbid diagnosis of major neurocognitive disorders. In the control group, we enrolled participants who were clinically diagnosed with mild or major neurocognitive disorders, Alzheimer’s type (MCI/AD) by DSM-5, with the same range of MMSE and age as MDD group. The participants with MCI/AD group were diagnosed based on medical history, clinical examination, basic and instrumental activities of daily living, MMSE or the Montreal Cognitive Assessment (MOCA), relevant laboratory and image investigations and standardized neuropsychological assessment. Participants in the control group were also excluded if they were comorbid with substance use disorder, other active physical conditions, known neurological symptoms or diseases such as brain injury or stroke. The study was approved by Research Ethics Committee of Taipei City Hospital (TCHIRB-10812017). All participants had given written informed consent before the enrollment.

## Measurements

### Demographic data and clinical course

Demographic data including age, gender, marital status, family history of psychiatric disorders, smoking habit, alcohol consumption, baseline physical comorbidities, age of onset, and year of education were collected at the time of enrollment. Medical characteristics including medications prescriptions and clinical course were gathered from medical records. The psychotropic medications that patients used at the time of assessment were recorded and converted in terms of defined daily dose (DDD). A DDD is a unit of measurement representing the assumed average maintenance dose per day for a drug used for its main indication in adults, which can be used for comparisons of drug consumption [29].

### Mood symptoms and cognitive measurements

Mood symptoms were assessed through the 15-item Geriatric Depression Scale (GDS-15), which had sufficient internal consistency reliability and retest reliability to support the use as a clinical instrument [30, 31]. Cognitive deficit was obtained through MMSE, using the 30-point version. The MMSE has been widely used to screen cognitive impairment in community settings and had renewed normative data in Taiwan [26, 32]. The examination had been carried out by trained researchers. All participants had not received assessments of MMSE during the past six months to prevent potential learning effect of repeated exposure.

### NfL levels analysis

A fasting venous blood sample of 10 mL was taken from each participant. The plasma was separated and stored at −80°C until the time of analysis. NfL levels were measured using Quanterix SiMoA^®^ assay, following the manufacturer’s standard procedures, which is a digital immunoassay with lower limit of detection being 0.104 pg/mL.

## Statistical analysis

We compared the demographic and clinical characteristics, as well as MMSE, GDS-15 scores, and NfL levels between the MDD patients and the MCI/AD patients. The chi-square and Student’s t tests were used for assessing categorical and continuous variables, respectively. Normality of data was assessed using the Kolmogorov-Smirnov test. For non-normally distributed data, we used non-parametric Wilcoxon rank sum test for analysis. Then, we used cluster analysis to separate participants into clusters by hierarchical method suggested tree diagram, according to their MMSE domains. Pearson correlation with adjustment of age was carried out to estimate the correlation between plasma NfL levels and cognitive domains in each cluster. All analyses were conducted using SPSS (version 22.0), and significance level was set at *p* < 0.05.

## Results

### Patient characteristics

Baseline assessments were completed in 39 patients from the MDD group and 15 patients from the MCI/AD group. The mean age of the MDD group (68.7 years) was significantly younger than that of the MCI/AD group (81.5 years, *p* < 0.001). In the MDD group, participants had higher rate of psychiatric family history (41% vs. 0%, *p* = 0.003), higher rate of current alcohol use habit (33% vs. 0%, *p* =0.010), and younger age of onset of disease (51.4 years vs. 77.3 years, *p* <0.001). They also had higher amounts of antidepressants use (0.83 DDDs vs. 0.55 DDDs, *p* =0.022) and benzodiazepines use (0.81 DDDs vs. 0.18 DDDs, *p* <0.001) as compared with the MCI/AD group. On the other hand, cognitive enhancing medications used in the MCI/AD group was significantly higher than that in the MDD group (0.64 DDDs vs. 0 DDD, *p* <0.001). These two groups showed no difference in gender, marital status, rate of current smokers, baseline physical comorbidities, education years, and amount of use of antipsychotics and mood stabilizers. The demographic and clinical characteristics were shown in Table 1.

**Table 1:** The demographic and clinical characteristics for participants

	MDD (*n*=39)	MCI/AD (*n*=15)	*P* value
Age, years (SD)	68.72 (6.04)	81.53 (5.71)	<0.001***
Gender Male, n (%)	8 (20.51)	3 (20.0)	0.966
Marriage: married or lived together, n (%)	27 (69.23)	7 (46.7)	0.124
With family psychiatry history, n (%)	16 (41.03)	0 (0.0)	0.003**
Current smoking, n (%)	4 (10.26)	0 (0.0)	0.273
Current alcohol use habit, n (%)	13 (33.0)	0	0.010*
With baseline physical comorbidity, n (%)	36 (92.3)	12 (80.0)	0.168
Onset of age, years (SD)	51.36 (14.00)	77.27 (4.85)	<0.001***
Education years, (SD)	10.64 (3.88)	10.53 (5.51)	0.936
DDD for psychoactive medications, (SD)			
First-generation antipsychotics	0.01 (0.03)	0	0.435
Second-generation antipsychotics	0.13 (0.425)	0.21 (0.389)	0.518
Mood stabilizers	0.04 (0.02)	0.02 (0.086)	0.247
Antidepressants	1.08 (0.83)	0.53 (0.550)	0.022*
Benzodiazepines	0.96 (0.806)	0.07 (0.179)	<0.001***
Cognitive enhancements	0	0.71 (0.641)	<0.001***

*MDD* major depressive disorder, *MCI* mild cognitive impairment, *AD* Alzheimer’s disease, *SD* standard deviation, *DDD* defined daily dose

^*^p < 0.05

^**^p < 0.01

^***^p < 0.001

### Cognitive manifestation between MDD and MCI/AD

In cognitive manifestation, participants in the MDD group had significantly higher scores in total MMSE score (27.1 vs. 22.3, *p* <0.001), orientation (9.4 vs. 7.6, *p* <0.001), attention and calculation (7.1 vs. 5.4, *p* <0.001), and comprehension (2.8 vs. 2.1, *p* =0.008). No significant difference was found in domains of memory, language or construction. MDD group also showed higher score in GDS-15 (5.9 vs. 3.2, *p* =0.032), which suggested more depressive symptoms noted in MDD than in MCI/AD. Meanwhile, plasma concentration of NfL was significantly higher in the MCI/AD group (28.95 vs. 15.69 pg/mL, *p* =0.012). The above results were listed in Table 2.

**Table 2:** The MMSE, GDS-15, and NfL levels between MDD and MCI/AD

	MDD (*n*=39)	MCI/AD (*n*=15)	*P* value
MMSE, Maximum score (SD)			
MMSE total, 30^a^	27.13 (2.56)	22.33 (3.42)	<0.001***
Orientation, 10	9.44 (0.94)	7.60 (1.60)	<0.001***
Attention and calculation, 8	7.10 (1.21)	5.40 (1.12)	<0.001***
Memory, 3	2.10 (0.99)	1.53 (1.25)	0.085
Language, 5	4.72 (0.69)	4.80 (0.86)	0.716
Comprehension, 3	2.79 (0.47)	2.06 (0.88)	0.008**
Construction, 1	0.97 (0.16)	0.93 (0.26)	0.573
GDS-15 (SD)^a^	5.94 (4.36)	3.20 (2.96)	0.032*
NfL levels (SD)	15.69 (8.81)	28.95 (17.04)	0.012*

*MMSE* Mini-Mental State Examination, *GDS-15* 15-item Geriatric Depression Scale, *NfL* neurofilament light chain, *MDD* major depressive disorder, *MCI* mild cognitive impairment, *AD* Alzheimer’s disease

^*^p < 0.05

^**^p < 0.01

^***^p < 0.001

^a^Analysis with non-parametric Wilcoxon rank sum test

### Cluster analysis and the association between NfL levels

All the participants were further divided into clusters according to their MMSE results (including 6 variables: orientation, attention/calculation, memory, language, comprehension and structure) using the hierarchical method suggested tree diagram. From the tree diagram, we applied two-step cluster analysis as the number of clusters was set to be two to six. For the distance measure, the log-likelihood criterion was used. Both Schwarz's Bayesian criterion (BIC) and the silhouette coefficient were used to compare cluster solutions. The silhouette coefficient was classified as poor (<0.2), fair (0.2-0.5), or good solution quality (>0.5). Fair or higher was considered acceptable clustering [33, 34]. In the current dataset, the two-cluster solution had the lowest BIC value (254) and a silhouette coefficient of 0.4. In cluster A (n=17), there were 6 participants from the MDD group and 11 participants from the MCI/AD group (35.3% and 64.7%, respectively). In cluster B (n=37), there were 33 participants from the MDD group and 4 participants from the MCI/AD group (89.2% and 10.8%, respectively). Therefore, we noticed part of the MDD patients presented cognitive deficits similar to that of patients with neurocognitive disorders (cluster A). Comparing the two clusters, the mean age of cluster A was older than that of cluster B (77.1 years vs. 70.0 years, *p* =0.003). GDS-15 scores showed no significant difference.

**Fig. 1:**
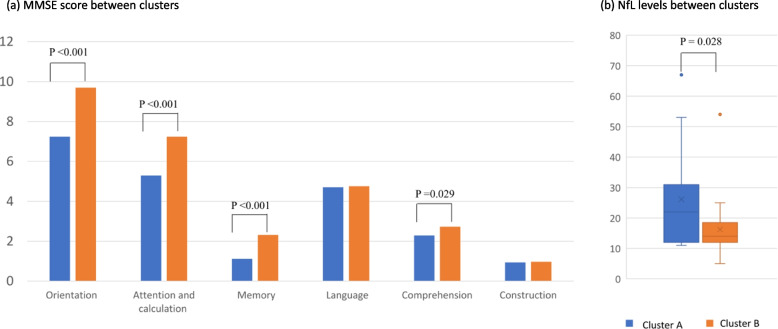
MMSE and NfL levels between clusters

Participants in cluster B obtained higher scores in total MMSE (27.7 vs. 21.6, *p* <0.001), orientation domain (9.7 vs. 7.2, *p* <0.001), attention and calculation domain (7.2 vs. 5.3, *p* <0.001), memory domain (2.3 vs. 1.1, *p* <0.001), and comprehension domain (2.7 vs. 2.3, *p* =0.029). Plasma levels of NfL in participants in cluster A is significantly higher than that of cluster B (26.21pg/mL vs. 16.95pg/mL, *p* =0.028) (Fig. 1). The cognitive profile difference between the two clusters was shown in Table 3. The main effect and interaction effect of diagnosis and cluster to both MMSE and NfL levels had been further confirmed by two-way ANCOVA (data not shown). In addition, in cluster A, the total MMSE score (r=-0.58277, *p* =0.0287) and the comprehension score (r=-0.71717, *p* =0.0039) were negatively correlated to plasma NfL levels, after adjusting for age. The same correlation pattern had not been observed in cluster B. The correlations between NfL levels and individual cognitive domain in each cluster were shown in Table 4.

**Table 3:** Cognitive profile difference between cluster groups

	Cluster A (*n*=17)	Cluster B (*n*=37)	*P* value
Age (SD)	77.12 (8.75)	70.05 (7.10)	0.003**
Educational years (SD)	9.12(4.05)	11.30 (4.35)	0.087
Diagnosis group MDD MCI/AD			<0.001***
	6	33	
	11	4	
GDS-15 (SD)^a^	5.59 (4.80)	5.02 (3.98)	0.656
NfL levels (SD)	26.21 (16.59)	16.95 (10.19)	0.028*
MMSE (SD)^a^	21.59 (2.50)	27.73 (1.84)	<0.001***
Orientation	7.24 (1.25)	9.70 (0.52)	<0.001***
Attention and calculation	5.29 (1.16)	7.24 (1.04)	<0.001***
Memory	1.12 (1.17)	2.32 (0.82)	<0.001***
Language	4.71 (0.85)	4.76 (0.68)	0.292
Comprehension	2.29 (0.92)	2.73 (0.51)	0.029*
Construction	0.94 (0.24)	0.97 (0.16)	0.574

*MDD* major depressive disorder, *MCI* mild cognitive impairment, *AD* Alzheimer’s disease, *GDS-15* 15-item Geriatric Depression Scale, *NfL* neurofilament light chain, *MMSE* Mini-Mental State Examination

^*^p < 0.05

^**^p < 0.01

^***^p < 0.001

^a^Analysis with non-parametric Wilcoxon rank sum test

**Table 4:** The correlation between NfL levels and cognitive domains in each cluster (adjusted age)

	Total MMSE	Orientation	Attention/ Calculation	Memory	Language	Comprehension	Structure
Cluster A							
NfL	-0.58277 *p* =0.0287*	-0.36598 *p* =0.1981	-0.08918 *p* =0.7618	-0.23707 *p* =0.4145	-0.00025 *p* =0.9993	-0.71717 *p* = 0.0039**	-0.00278 *p* =0.9925
Cluster B							
NfL	-0.05822 *p* =0.7642	0.11068 *p* =0.5676	0.02806 *p* =0.8851	-0.28356 *p* =0.1361	-0.07125 *p* =0.7134	0.17088 *p* =0.3755	0.16213 *p* =0.4007

*NfL* neurofilament light chain

^*^p < 0.05

^**^p < 0.01

## Conclusions

What core cognitive deficits in MDD might naturally develop into clinical dementia is still poorly understood. Moreover, clinical diagnosis of MDD and dementia are all with high heterogeneity and imply different etiology [35]. In current study with cluster analysis, our results demonstrated that a proportion of older patients with MDD presented cognitive deficits that was similar to that of MCI/AD. These findings were consistent with previous evidence that amnestic cognitive deficits in late life of MDD were more like to become AD compared with non-amnestic type [17]. Furthermore, plasma NfL levels in this cluster of patients were negatively correlated to their total MMSE cognitive performance, especially in comprehension domain. The elevation of NfL levels in older adults with MDD might imply an underlying neuroaxonal pathology, which might indicate an increased risk of progression to dementia.

Previous studies about NfL levels in MDD patients have revealed inconsistent findings. Some of them have shown increased peripheral NfL concentration in patients with MDD compared with health control; however, others did not find the difference, especially in cognitively unimpaired MDD sample [36–38]. Two studies have further reported the association of NfL levels with executive function and processing speed in MDD patients [37, 39]. On the other hand, the increased NfL levels seemed to be related to higher risk of depression in patients with ischemic stroke [40] and Parkinson’s disease [41]. Subgroup of MDD patients can share the same pathophysiology from depression to dementia [42]. Early life depression can act as a risk factor for later life dementia, and that late onset depression can be seen as a prodrome to dementia [42]. Studies suggested that genetic factors, decreased hippocampal sizes, metabolic co-morbidities, obesity, inflammatory status, and unhealthy life style such as lack of exercises might contribute part of the underlying mechanism of impaired cognitive function in MDD, which might lead to increased risk of incident dementia [43–45].

In depressive state, executive function was one of the most presented deficits, especially in older depressed population [46, 47]. Furthermore, the level of executive deficit seemed to be associated with the severity of depression [46]. However, the relationship between the clinical characteristics of MDD (such as number of major depressive episodes and subtypes of MDD) and the severity of cognitive impairment remained inconsistent [48]. Other evidences also revealed impairment of episodic memory and processing speed, which might mediate executive function partially, in older MDD patients [46, 47, 49, 50]. Some of them recovered along with the remission of depressive symptoms but some progressed to dementia. Steffens et al. reported about 15% of older depressed adults might progress to dementia [51]. Potter et al. found depressed older adults with deficits in the domains of encoding memory and executive function present higher risk for progression to dementia [52]. Therefore, the difference in manifestation of cognitive domains may provide a hint for clinicians to identify older adults with depression who carry a higher risk of incident dementia. In our study, subgroup of MDD patients clustered with majority of MCI/AD in terms of their impairment on orientation, attention/calculation, memory and comprehension domains through MMSE. In the above cluster, plasma NfL levels showed a negative correlation with total MMSE score and comprehension score. The comprehension domain in MMSE may represent a combined ability of verbal comprehension, short-term memory, and executive function, which are the typically impaired domains found in the process of AD (Hugo 2014, Bondi 2017). The non-significant difference between clusters in language domain and construction domain may be due to the ceiling effect, because these domains usually decline in the later course of AD [53–56].

## Strengths and limitations

The major strength of current study is the use of cluster analysis, which dissected the MDD group and the MCI/AD group cross the diagnoses. Therefore, we overcome the heterogeneity in clinical diagnoses of MDD and dementia patients and subgroup them by similar cognitive deficit profiles. There were several limitations in this study. First, our sample size was relatively small, thus limited the power to examine effects among potential variables, such as disease course and medications, or analysis solely for MDD patients in cluster A. Second, our participants were recruited from a tertiary psychiatry hospital where patients may have more severe degree of illnesses. For instance, participants in the AD group might present more severe behavioral and psychological symptoms of dementia. In addition, this potentially biased older MDD sample may limit the generalizability of our findings to the whole older MDD population, for example, the percentage of MDD with similar cognitive deficit with MCI/AD. Third, our study was cross-sectional design. There was a lack of follow-up data regarding changes in participants’ cognitive performance or levels of NfL. Thus, causal relationship could not be proven. Fourth, we did not separate participants in the MDD group into early-onset and late-onset group because of limited sample size. Patients with early-onset MDD might present different clinical manifestation as compared with late-onset MDD, and there might be differences in risk and mechanism of developing cognitive impairment and incident dementia [57–59]. Fifth, multiple comparison should be considered since we compared 6 domains in MMSE. The finding in the domain of comprehension was still significant if we corrected *p* value with Bonferroni method. Finally, we have chosen MCI/AD as active control in our sample; however, there was absence of a normal control group for comparison in current study. In addition, further studies investigating older MDD to develop other common types of degenerative neurocognitive disorders, such as frontal-temporal lobe dementia, or Parkinson’s disease dementia are warrant.

## Summary and recommendations

The concentration of peripheral NfL showed negative correlation with cognitive performance among older MDD patients who clustered with cognitive deficit of MCI/AD. Therefore, NfL could be a potential marker to predict older MDD patients to develop cognitive decline in domains that were typically found in Alzheimer’s disease. This might provide a chance of early recognition and intervention in these patients. Further longitudinal studies, and within MDD cluster analysis and also investigations for other types of dementia are required to validate our findings for clinical implications.

## Data Availability

The datasets used and analysed during the current study are available from the corresponding author on reasonable request.
